# Chronic Disease Risk Factors Among Alaska Native and American Indian People, Alaska, 2004-2006

**Published:** 2010-06-15

**Authors:** Diana G. Redwood, Anne P. Lanier, Janet M. Johnston, Elvin D. Asay, Martha L. Slattery

**Affiliations:** Alaska Native Tribal Health Consortium Community Health Services; Alaska Native Tribal Health Consortium, Anchorage, Alaska; Alaska Native Tribal Health Consortium, Anchorage, Alaska; Alaska Native Tribal Health Consortium, Anchorage, Alaska; University of Utah, Salt Lake City, Utah

## Abstract

**Introduction:**

The Alaska Education and Research Towards Health (EARTH) Study is being conducted to determine the prevalence of clinically measured chronic disease risk factors in a large population of American Indian/Alaska Native people (AI/AN). We report these estimates and compare them with those for the overall US population, as assessed by the National Health and Nutrition Examination Survey (NHANES).

**Methods:**

We measured blood pressure, height, weight, and fasting serum lipids and glucose in a prospective cohort of 3,822 AI/AN participants who resided in Alaska during 2004 through 2006. We categorized participants as having chronic disease risk factors if their measurements exceeded cutoffs that were determined on the basis of national recommendations. We analyzed the prevalence of risk factors by sex and age and compared the age-adjusted prevalence with 1999-2004 NHANES measurements.

**Results:**

EARTH participants were significantly more likely than NHANES participants to be overweight or obese and to have impaired fasting glucose, low high-density lipoprotein cholesterol, high low-density lipoprotein cholesterol, and hypertension. The prevalence of high total cholesterol and triglycerides was not significantly different between the 2 study populations.

**Conclusion:**

We provide baseline clinical measurements for chronic disease risk factors for a larger study sample than any previous study of AI/AN living in Alaska. The prevalence of most risk factors measured exceeded national rates. These data can be used to tailor health interventions and reduce health disparities.

## Introduction

Alaska Native people are a heterogeneous population; more than 200 federally recognized tribes are widely dispersed in rural and urban areas across the state. The prevalence of chronic diseases is higher among tribal communities than among other US racial/ethnic minority populations, and the associated mortality is higher than among US non-Hispanic whites (1,2). Alaska Native people experience lower mortality from diabetes and heart disease than do other US racial/ethnic minority populations but higher mortality from cerebrovascular diseases and malignant neoplasms (2). Heart disease, cerebrovascular disease, chronic obstructive pulmonary disease, chronic liver disease, and diabetes are 5 of the 10 leading causes of death among Alaska Native people (1). Additionally, the prevalence of risk factors such as tobacco use and obesity is higher among Alaska Native than non-Native Alaska residents (3).

American Indian and Alaska Native people (AI/AN) are generally not included in national surveys that monitor health status, or the numbers are too small to draw meaningful conclusions. Data, when available, are typically from telephone surveys and are self-reported. Few studies have collected data for AI/AN from clinical measurements, and those that have focused largely on a specific disease, age group, or geographic area (4-6). No data are available to indicate the prevalence of measured clinical risk factors from a large population of AI/AN. Thus, it is unclear whether the prevalence of selected clinical risk factors is the same for AI/AN as for other populations.

The Alaska Education and Research Towards Health (EARTH) Study is a prospective cohort study of predominately Alaska Native people (95% AN, 5% AI) who reside in the state. It is designed to examine the effects of lifestyle and clinical risk factors on the development of chronic diseases. The objective of our analysis was to report measured clinical data for blood pressure, height, weight, and fasting serum lipids and glucose among EARTH participants and to compare the prevalence of elevated risk in this population with that of all US racial/ethnic populations assessed by the National Health and Nutrition Examination Survey (NHANES).

## Methods

### Study population

We have described the methods of the EARTH Study in detail elsewhere (7). We recruited participants from southwest (Yukon-Kuskokwim Delta), southeast (Panhandle), and southcentral (Anchorage area) Alaska. Eligibility criteria included being aged 18 years or older, being AI/AN and eligible for health care through the Indian Health Service, residing in the study area, and being able to provide informed consent. Methods of recruitment included presentations to tribal groups and health care providers, informational tables staffed by study personnel at community events, house-to-house recruiting, brochures and flyers in public locations, and public service announcements on local radio and in newspapers. The Alaska Area Institutional Review Board, the Indian Health Service Institutional Review Board, the regional tribal health organizations, and the tribal councils of participating study communities approved the study.

We summarize data collected from 3,822 participants in 26 Alaska communities from March 2004 through August 2006. We asked pregnant women and chemotherapy patients to participate at a later date. On the basis of 2000 AI/AN census data for each community, participation ranged from 2% to 49% of eligible adults; the median participation rate was 29%.

### Measurement and outcomes

EARTH participants completed self-administered and interviewer-administered questionnaires on demographics, diet, physical activity, lifestyle and cultural practices, environmental exposures, cancer-screening practices, medical and reproductive history, and family history of chronic diseases. Computer-assisted questionnaires on touch-screen panels were used with an audio version of the questionnaires in English or Yupik (8). In the questionnaires, we asked participants if a health care provider had ever told them that they had specific chronic diseases. All participants were included in the analysis even if they had a self-reported medical history of hypertension, diabetes, or dyslipidemia.

We measured serum lipids and glucose, seated blood pressure, and standing height, weight, and waist circumference. We report the measurement methods in detail elsewhere (7,9,10). We asked study participants to fast for 9 hours before their study visit to meet requirements for triglyceride and glucose measurements. Only participants who reported fasting for 9 hours or more were measured for serum lipids and glucose. We gave participants who enrolled but had not fasted the option of completing the visit except for the blood test and returning at a later date to complete the blood test.

We based the clinical risk factor categories on recommendations from the National Cholesterol Education Program, the American Diabetes Association, and the Joint National Committee on Prevention, Detection, Evaluation, and Treatment of High Blood Pressure (11-13). Specifically, we used the following definitions: hypertension (≥140 mm Hg systolic or ≥90 mm Hg diastolic), prehypertension (120-139 mm Hg systolic or 80-89 mm Hg diastolic), high serum cholesterol (≥200 mg/dL), high low-density lipoprotein (LDL) cholesterol (≥130 mg/dL), low high-density lipoprotein (HDL) cholesterol (<40 mg/dL), high triglycerides (≥150 mg/dL), overweight (body mass index [BMI] 25.0-29.9 kg/m^2^), obesity (BMI ≥30.0 kg/m^2^), extreme obesity (BMI ≥40.0 kg/m^2^), abdominal obesity (waist circumference >102 cm for men and >88 cm for women), and high fasting plasma glucose (3 risk categories: 100-109 mg/dL, 110-125 mg/dL, and >125 mg/dL).

### Statistical analysis

We computed basic summary statistics to provide an overview of the study sample compared with all AI/AN in Alaska (2000 US census data) (14,15). To determine the prevalence of the selected clinical risk factors in the study population, we transformed continuous data into risk categories and calculated 95% confidence intervals (CIs). We determined the percentage of participants who exceeded cutoff levels separately for men and women aged 18 to 39 years, 40 to 59 years, and 60 years or older. Age and sex differences for all risk factors were calculated using logistic regression, and all analyses were conducted using SPSS version 16 for Windows (IBM, Chicago, Illinois). Significance was set at *P* < .05.

For comparison with NHANES, only data for EARTH participants aged 20 years or older were included (n = 3,568). EARTH data were age-adjusted by the direct method to 2000 census data estimates using 5 age groups: 20 to 34, 35 to 44, 45 to 54, 55 to 64, and 65 years or older (16). We obtained 1999-2004 NHANES data for all US racial/ethnic groups from published sources for comparison (16-20). In addition to testing for nonoverlapping 95% CIs, we used the methods described by Dever (18) for calculating critical values to test for difference in rates between the observed rate (EARTH) and a standard rate (NHANES). All differences that had nonoverlapping CIs were significantly different according to the Dever method.

## Results

Most participants were women (61%), had at least a high school education (77%), were unmarried (57%), were unemployed (55%), and rated their health status as good or better (75%) (Table 1). More than 33% of participants reported speaking either their native language only or both their native language and English in the home. The most common ethnicities reported were Yupik, Cupik, or Inupiaq Eskimo (62%), followed by Southeast Alaska Indian (Haida, Tlingit, Tsimshian) (29%), Athabaskan (10%), Aleut (9%), and American Indian (5%) (participants could report more than 1 ethnicity). Eight percent of participants reported that they were of native descent, tribe unknown. Demographic characteristics were similar to those of census-generated estimates for AI/AN residing in Alaska, but EARTH participants reported lower income, higher unemployment, and lower health status.

The most common risk factor overall among men was prehypertension (49%) (Table 2). For men aged 40 to 59 years, high serum cholesterol was the most common risk factor (49%), and for men aged 60 years or older, abdominal obesity was the most common risk factor (57%). The most common risk factor among women was abdominal obesity (70%); this was true across all age groups but was highest among women aged 60 years or older.

The age-adjusted odds of hypertension, prehypertension, high LDL cholesterol, overweight, and the 100 to 109 mg/dL glucose risk category were significantly higher among men than women (Table 3). The higher risk categories for blood glucose (≥110 mg/dL) were not significantly associated with sex after adjusting for age. The odds of obesity and extreme obesity were significantly higher among women than men. For most risk factors, sex-adjusted prevalence increased significantly with age. The rates of overweight and extreme obesity were not significantly associated with age after adjusting for sex.

**Figure 1:**
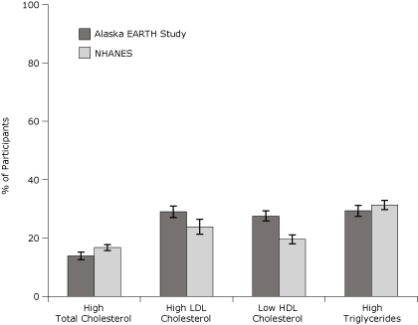
Age-adjusted prevalence of dyslipidemia for Alaska EARTH Study participants, 2004-2006, compared with Alaska NHANES participants, 1999-2004. Error bars represent 95% confidence intervals. Direct age standardization is based on the 2000 census standard population for adults aged 20 years or older using age groups 20 to 34 years, 35 to 44 years, 45 to 54 years, 55 to 64 years, and 65 years or older. Clinical risk factor categories for EARTH Study participants are based on recommendations from the National Cholesterol Education Program (11). Clinical risk factor categories for NHANES participants are based on *Health, United States, 2007* (16), Hyre et al (19), and Kuklina et al (20). Abbreviations: EARTH, Education and Research Towards Health; NHANES, National Health and Nutrition Examination Survey; LDL, low-density lipoprotein; HDL, high-density lipoprotein.

**Figure 2:**
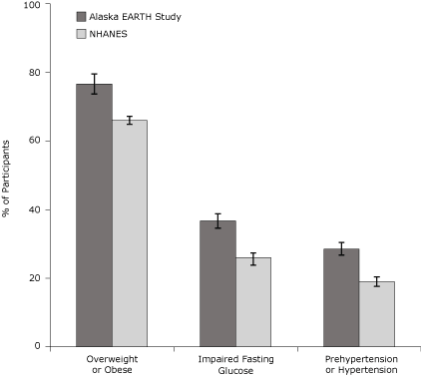
Age-adjusted prevalence of clinical risk factors for Alaska EARTH Study participants, 2004-2006, compared with Alaska NHANES participants, 1999-2004. Error bars represent 95% confidence intervals. Direct age standardization is based on the 2000 census standard population for adults aged 20 years or older using age groups 20 to 34 years, 35 to 44 years, 45 to 54 years, 55 to 64 years, and 65 years or older. Clinical risk factor categories for EARTH Study participants are based on recommendations from the American Diabetes Association and the Joint National Committee on Prevention, Detection, Evaluation, and Treatment of High Blood Pressure (12,13). Clinical risk factor categories for NHANES participants are based on *Health, United States, 2007* (16) and Cowie et al (17). Abbreviations: EARTH, Education and Research Towards Health; NHANES, National Health and Nutrition Examination Survey.

The prevalence of low HDL and high LDL cholesterol was higher among EARTH participants than among NHANES participants but did not differ significantly for high total cholesterol or triglycerides (Figure 1). Additionally, the prevalence of overweight, obesity, impaired fasting glucose, and hypertension were significantly higher among EARTH participants than among NHANES participants (Figure 2). The prevalence of overweight and obesity among AI/AN women in the EARTH Study was particularly high (78%) compared with all races/ethnicities from NHANES (66%).

**Table:** 

**Risk Factor**	**Alaska EARTH Study, % (95% CI)**	**NHANES, % (95% CI)**
High total cholesterol	13.8 (12.6- 15.1)	16.7 (15.7-17.7)
High LDL cholesterol	29.0 (27.0- 30.9)	23.8 (21.3- 26.4)
Low HDL cholesterol	27.5 (25.8- 29.3)	19.5 (17.9- 21.1)
High triglycerides	29.3 (27.4- 31.2)	31.3 (29.7- 32.9)

**Table:** 

**Risk Category**	**Alaska EARTH Study, % (95% CI)**	**NHANES, % (95% CI)**
Overweight	76.6 (73.7-79.5)	66.0 (64.8-67.2)
Impaired fasting glucose	36.7 (34.6-38.8)	26.0 (23.8-28.4)
Prehypertension or hypertension	28.5 (26.7-30.4)	19.0 (17.6-20.4)

## Discussion

This study provides detailed results of selected clinical risk measurements from almost 4,000 AI/AN adults living in both rural and urban areas of Alaska. We document high prevalence of chronic disease risk factors in this population and differences between this population and the US overall population on the basis of NHANES data. The prevalence of most risk factors in the Alaska study group increased with age and was more common among men than women, except for overweight and obesity.

The overall prevalence of hypertension (men, 13%; women, 11%) was the lowest among the risk factors measured in this study. However, 28% to 49% of participants had prehypertension. The prevalence of hypertension among EARTH participants was significantly higher than among NHANES participants for all races/ethnicities. The Strong Heart Study reported that in nondiabetic American Indian participants aged 45 to 74 years, prehypertension increased the likelihood of cardiovascular events by 80% compared with their normotensive counterparts (21). Similar results were reported by the Framingham Heart Study (22).

The overall prevalence of high cholesterol among EARTH participants was 40%. The prevalence of low HDL and high LDL cholesterol among EARTH participants was higher than among NHANES participants. Historically, Alaska Natives subsisted on wild foods such as fish and marine mammals, which provided healthful fats. However, most Alaska Natives are now more sedentary and have transitioned to a mixed traditional and Western diet, and the high prevalence of dyslipidemia is likely to continue (23,24).

The prevalence of overweight and obesity also was high among EARTH participants (women, 78%; men, 68%). A similar trend held for abdominal obesity. Although obesity was virtually nonexistent in Alaska before European explorations (25), studies of Alaska Natives in remote villages in the 1960s through 1980s documented a steady increase in the percentage of the population aged 40 years or older who weighed 14 kg (30 lb) above the average for whites of the same age, height, and sex (26). These trends are similar to other AI/AN populations living in the contiguous United States. A review of data from the Indian Health Service Diabetes Care and Outcomes Audit found that BMI was consistently higher among women than men in all age groups and among younger AI/AN adults and that the prevalence of extreme obesity was increasing (27). Among EARTH participants, the prevalence of extreme obesity was also higher in women than men, particularly younger women.

Short-term studies indicate that approximately 25% of people with impaired fasting glucose (IFG) progress to diabetes (28), and with longer observation, up to 70% appear to develop diabetes (29). NHANES age-adjusted data indicate the prevalence of IFG to be approximately 26% nationally (17), whereas EARTH age-adjusted IFG prevalence was 37%. As found in other studies, the prevalence of IFG increased with age among study participants and was higher among men than women.

This study has several limitations. First, analysis was based on a convenience sample that included more women than men. However, when possible, we report sex-specific data. The age, education, and marital status of participants were similar to those reported in the 2000 census for AI/AN living in Alaska. Second, lipids and glucose were measured with a fingerstick blood sample using the Cholestech LDX (Cholestech, Hayward, California), a method that may not be directly comparable with the NHANES laboratory method. However, studies have shown good correlation of lipid values measured by the Cholestech LDX and those from serum obtained from venous blood samples (10,30). In addition, the EARTH Study adhered to quality control standards, including staff training, procedure manuals, logs, and site visits, to assure consistent data collection. Finally, the EARTH Study did not assess the use of medications for hypertension and dyslipidemia, which may have influenced lipid and blood pressure values.

We have documented the high prevalence of chronic disease risk factors among AI/AN living in Alaska and noted differences compared with the general US population. Measurements for a substantial proportion of study participants were outside of normal ranges but below the cutoff for clinical diagnosis. Studies are needed to determine the effect of these risk factors on chronic disease incidence in AI/AN populations. These data can be used to prioritize health promotion and disease prevention activities at the local and regional levels to reduce health disparities among Alaska Native populations.

## Figures and Tables

**Table 1:** Characteristics of AI/AN Alaska Residents Who Participated in the EARTH Study, 2004-2006, Compared With AI/AN Alaska Residents Overall, US Census, 2000

**Characteristic**	EARTH Study, No. (%) N = 3,822	Census^a^, No. (%) N = 119,331
**Region^b^**		
Southcentral	1,394 (37)	36,428 (51)
Southeast	887 (23)	15,079 (21)
Southwest	1,541 (40)	20,226 (28)
**Age, y^c^**		
18-39	1,874 (49)	37,235 (52)
40-59	1,517 (40)	24,816 (35)
≥60	431 (11)	8,913 (13)
**Sex^c^**		
Men	1,502 (39)	60,212 (51)
Women	2,320 (61)	59,119 (49)
**Marital status^d^**		
Married or living as married	1,630 (43)	30,705 (39)
Separated, divorced, or never married	2,177 (57)	48,118 (61)
**Education^c^**		
High school diploma or more	2,933 (77)	43,469 (74)
**Employment status^e^**		
Employed or self-employed	1,717 (45)	45,734 (60)
**Annual household income, $^f^**		
≤15,000	1,343 (41)	6,536 (20)
15,001-25,000	526 (16)	5,178 (16)
25,001-35,000	446 (14)	4,403 (14)
35,001-50,000	444 (14)	5,074 (16)
>50,000	505 (15)	10,904 (34)
**Language spoken at home^g^**		
Non-English or both	1,262 (33)	31,130 (29)
English only	2,542 (67)	76,435 (71)
**Self-reported health status^h^**		
Excellent, very good, or good	2,864 (75)	2,403 (87)
Fair or poor	952 (25)	400 (13)
**Ethnicity^i^**		
Eskimo	2,357 (62)	NA
Southeast Alaska Indian	1,089 (29)	NA
Athabaskan	390 (10)	NA
Aleut	323 (9)	NA
American Indian	205 (5)	NA
Other Native (tribe unknown)	285 (8)	NA

Abbreviations: AI/AN, American Indian/Alaska Native people; EARTH, Education and Research Towards Health; NA, not assessed.

a Census 2000 data for Alaska (14). Totals do not equal 119,331 for all categories because of missing responses.

b Census 2000 data for the corresponding Alaska Native Regional Corporations [ANRC]) Southcentral: Cook Inlet ANRC; Southeast: Sealaska ANRC; Southwest: Calista ANRC (14).

c Census 2000 data for population aged 18 years or older (14)

d Census 2000 data for population aged 15 years or older (14).

e Census 2000 data for population aged 16 years or older (14).

f Census 2000 data for households. EARTH data were missing 555 responses for this variable (14).

g Census 2000 data for population aged 5 years or older (14).

h Behavioral Risk Factor Surveillance System 2005, Alaska, all races/ethnicities (15).

i Percentages do not total 100 because participants could choose more than 1 ethnicity.

**Table 2:** Prevalence of Clinical Risk Factors for Chronic Disease, by Age and Sex, Among Alaska Native and American Indian People, EARTH Study, Alaska, 2004-2006

Risk Factor^a^	Men, % (95% CI)			

	Age 18-39 y n = 742	Age 40-59 y n = 610	Age ≥60 y n = 150	All Ages n = 1,502
**Hypertension**	10 (8-12)	14 (11-16)	22 (15-29)	13 (8-17)
**Prehypertension**	55 (52-59)	45 (41-49)	38 (31-46)	49 (46-53)
**High total cholesterol**	31 (28-35)	49 (45-53)	48 (40-56)	40 (36-44)
**High LDL cholesterol**	27 (24-31)	37 (33-41)	37 (28-45)	32 (27-36)
**Low HDL cholesterol**	27 (24-31)	20 (17-23)	24 (17-30)	24 (19-28)
**High triglycerides**	25 (22-28)	29 (26-33)	29 (21-36)	27 (22-31)
**Abdominal obesity**	28 (25-31)	41 (37-45)	57 (49-65)	36 (32-40)
**BMI, kg/m^2 ^ b**				
25.0-29.9	34 (31-37)	38 (34-41)	36 (28-44)	36 (32-40)
≥30.0	29 (25-32)	33 (29-37)	43 (35-51)	32 (28-36)
≥40.0	5 (4-7)	6 (4-7)	6 (2-10)	6 (1-10)
**Fasting glucose, mg/dL**				
100-109	26 (23-30)	31 (27-34)	26 (19-33)	28 (24-32)
110-125	7 (6-9)	13 (11-16)	21 (15-28)	11 (6-16)
>125	3 (2-5)	7 (5-9)	13 (7-18)	6 (1-11)
**Risk Factor** a	**Women, % (95% CI)**			

	**Age 18-39 y n = 1,132**	**Age 40-59 y n = 907**	**Age ≥60 y n = 281**	**All Ages n = 2,320**
**Hypertension **	5 (3-6)	13 (11-16)	29 (24-34)	11 (7-15)
**Prehypertension**	22 (19-24)	32 (29-35)	40 (34-45)	28 (24-31)
**High total cholesterol**	26 (23-29)	51 (48-55)	63 (57-69)	40 (37-44)
**High LDL cholesterol**	14 (12-16)	28 (25-31)	39 (33-45)	22 (19-26)
**Low HDL cholesterol**	13 (11-15)	7 (5-9)	11 (7-14)	11 (7-14)
**High triglycerides**	25 (23-28)	32 (29-35)	34 (28-39)	29 (26-32)
**Abdominal obesity**	65 (62-68)	74 (71-77)	80 (75-84)	70 (68-72)
**BMI, kg/m^2 ^ b**				
25.0-29.9	29 (26-31)	30 (27-33)	31 (26-37)	29 (26-33)
≥30.0	47 (44-50)	51 (48-54)	52 (46-58)	49 (46-52)
≥40.0	11 (9-13)	11 (9-13)	8 (4-11)	11 (7-14)
**Fasting glucose, mg/dL **				
100-109	19 (17-22)	22 (19-25)	28 (23-34)	21 (18-25)
110-125	6 (4-7)	13 (11-15)	18 (13-22)	10 (6-14)
>125	3 (2-4)	7 (5-8)	7 (4-10)	5 (1-9)

Abbreviations: EARTH, Education and Research Towards Health; CI, confidence interval; LDL, low-density lipoprotein; HDL, high-density lipoprotein; BMI, body mass index.

a Clinical risk factor categories based on recommendations from the National Cholesterol Education Program, the American Diabetes Association, and the Joint National Committee on Prevention, Detection, Evaluation, and Treatment of High Blood Pressure (11-13).

b BMI categories are not mutually exclusive. All of the participants who appear in the ≥40 category also appear in the ≥30 category.

**Table 3:** Odds of Clinical Risk Factors for Chronic Disease, by Age and Sex, Among Alaska Native and American Indian People, EARTH Study, Alaska, 2004-2006

**Risk Factor^a^**	OR^b^ for 1-Year Increase in Age (95% CI)	OR^c^ for Men Compared With Women (95% CI)
**Hypertension**	1.03 (1.03-1.04)	1.66 (1.43-1.94)
**Prehypertension**	1.01 (1.01-1.02)	2.45 (2.12-2.85)
**High total cholesterol**	1.04 (1.03-1.04)	1.04 (0.91-1.20)
**High LDL cholesterol**	1.03 (1.02-1.03)	1.71 (1.46-2.01)
**Low HDL cholesterol**	0.99 (0.98-0.99)	0.68 (0.59-0.80)
**High triglycerides**	1.01 (1.01-1.02)	0.98 (0.84-1.15)
**Abdominal obesity**	1.03 (1.02-1.03)	0.24 (0.21-0.27)
**BMI, kg/m^2 ^ d**		
25.0-29.9	1.00 (1.00-1.00)	1.32 (1.15-1.52)
≥30.0	1.01 (1.01-1.02)	0.50 (0.43-0.57)
≥40.0	1.00 (1.00-1.00)	0.50 (0.39-0.65)
**Fasting glucose, mg/dL**		
100-109	1.01 (1.01-1.02)	1.46 (1.25-1.71)
110-125	1.03 (1.03-1.04)	1.20 (0.96-1.50)
>125	1.03 (1.02-1.04)	1.27 (0.94-1.71)

Abbreviations: EARTH, Education and Research Towards Health; OR, odds ratio; CI, confidence interval; LDL, low-density lipoprotein; HDL, high-density lipoprotein; BMI, body mass index.

a Clinical risk factor categories based on recommendations from the National Cholesterol Education Program, the American Diabetes Association, and the Joint National Committee on Prevention, Detection, Evaluation, and Treatment of High Blood Pressure (11-13).

b Adjusted for sex. All values except those for BMI 25.0-29.9 and BMI ≥40.0 were significant at *P* < .001 (χ^2^ test).

c Adjusted for age. All values except those for high total cholesterol, high triglycerides, fasting glucose 110-125 mg/dL, and fasting glucose >125 mg/dL were significant at *P* < .001 (χ^2^ test ).

d BMI categories are not mutually exclusive. All of the participants who appear in the ≥40 category also appear in the ≥30 category.
